# Elastic Properties and Hardness of Mixed Alkaline Earth Silicate Oxynitride Glasses

**DOI:** 10.3390/ma15145022

**Published:** 2022-07-19

**Authors:** Sharafat Ali

**Affiliations:** Department of Built Environment and Energy Technology, School of Engineering, Linnæus University, SE-351 95 Växjö, Sweden; sharafat.ali@lnu.se

**Keywords:** oxynitride glass, mixed modifiers, high nitrogen, hardness, elastic moduli, Poisson’s ratio

## Abstract

The incorporation of nitrogen as a second anion species into oxide glasses offers unique opportunities for modifying glass properties via changes in glass polymerization and structure. In this work, the compositional dependence of elastic properties and the nanoindentation hardness of mixed alkaline-earth silicate oxynitride glasses containing a high amount of nitrogen (>15 at.%, c.a. 35 e/o) were investigated. Three series of silicon oxynitride glass compositions *AE–Ca–Si–O–N* glasses (where *AE = Mg*, *Sr*, and *Ba*) having varying amounts of modifiers were prepared using a new glass synthesis route, in which a precursor powder of metal hydrides was used. The obtained glasses contained high amounts of *N* (19 at.%, c.a. 43 e/o) and modifier cations (26 at.%, c.a. 39 e/o). *Mg–Ca–Si–O–N* glasses had high values of nanohardness (12–16 GPa), along with a reduced elastic modulus (130–153 GPa) and Young’s modulus (127–146 GPa), in comparison with the Sr–Ca- and Ba–Ca-bearing oxynitride glasses. Both the elastic modulus and the nanohardness of *AE–Ca–Si–O–N* glasses decreased with an increase in the atomic number of the *AE* element. These property changes followed a linear dependence on the effective cation field strength (*ECFS*) of the alkaline earth (*AE*) modifier, according to their valences and ionic radii. No mixed alkaline-earth effect was observed in the current investigation, indicating that the properties were more dictated by the nitrogen content.

## 1. Introduction

Oxynitride glasses have attracted accelerating interest over the last few decades in both academia and industry because their mechanical, thermal, and optical properties are tunable with changing composition, especially with respect to the N/O ratio and various alkaline earth or rare earth modifying ions. Most of the silicon-based oxynitride glasses prepared using the traditional synthesis route, in which a modifier is introduced to the glass network in its oxide form, have a rather limited amount of nitrogen (<7 at.%) in the glass network [1,2,3,4,5,6,7,8,9]. However, the glasses prepared using pure metal or metal hydride, Si_3_N_4_, and SiO_2_ powders contain a very high amount of nitrogen (>65 e/o) and modifier [10,11,12,13,14,15,16,17]. The glasses in the present study were prepared using metal hydride as a precursor.

Oxynitride glasses have been synthesized in a number of *M–Si–(Al)–O–N* systems (where *M* = alkaline earth or rare earth metal). From these studies, it was observed that the glass transition temperature, hardness, elastic modulus, resistance to vitrification, refractive index, viscosity, and dielectric constant increase systematically with increasing N/O ratio [12,18,19,20,21,22]. A similar trend was also observed in metal-containing *Si–(Al)–O–N* thin films [23,24,25,26,27,28]. The variation of properties with the N content is due to the increased crosslinking with the glass structure due to the three-coordinated N atoms. However, the observed changes in the properties in oxynitride glasses and thin films are explained not only by increasing the nitrogen content but possibly also by the modifier element type and its concentration.

Studies on both oxide and oxynitride glasses revealed that the properties of mixed modifier glasses vary linearly by changing the concentration of one modifier at the expense of another modifier. Very few studies have been conducted on mixed-modifier cation oxynitride glasses [29,30], and these studies were more concerned with the effects of nitrogen substitution for oxygen rather than the effect of mixed cation modifier compositions or ratios. Pomeroy and Abbas et al. [31,32] and Sharafat et al. [33] reported the properties of glasses in rare earth silicon (aluminum) oxynitride and alkaline earth silicon oxynitride systems, respectively, containing mixed modifiers and having a constant ratio of *Si–(Al)–O–N*. In a previous study, we systematically investigated the effects of alkaline earth elements on the physical (density and molar volume), thermal (glass transition and crystallization temperatures), mechanical (hardness), and optical (refractive index) properties of an *AE–Ca–Si–O–N* system with constant *Si–O–N* composition [33]. It was found that the thermal properties and hardness increased sturdily upon increasing the effective cation field strength (*ECFS*) of the modifier ions. Furthermore, the variations in refractive index and density are attributed to the different atomic weights of the modifier ions. The present work comprises the first report on the elastic properties of mixed modifier alkaline earth silicon oxynitride glasses containing a high nitrogen content (>15 at.%).

Elasticity is an intrinsic property of materials and a crucial factor in material development and engineering design. The elastic properties of glass give an inclusive view of material stiffness, and they reflect both the network connectivity and the interatomic potential; furthermore, they are used to determine the thermal shock resistance and fracture toughness of glass [34,35]. Currently, there is increasing interest in the development of new glass compositions to achieve a high elastic modulus and low density for advanced applications. In comparison with oxide glasses, little is known about the effects of composition on the elastic properties of oxynitride glasses. The field strength of network-modifying cations also strongly influences the properties of the (oxy)nitride glasses. In the present paper, we investigate the correlations between the effective cation field strength of the network modifier ions and physical properties, e.g., hardness, elastic modulus, and Poisson’s ratio. For this, nitrogen-rich glasses (typically >15 at.%) in the mixed alkaline earth Si–O–N system were studied using nanoindentation and ultrasonic echography methods. Furthermore, the property variations with respect to the effect of mixed modifier content in the glasses were investigated.

## 2. Experimental Procedure

Three series of glasses with approximately the same silicon, oxygen, and nitrogen contents in an *AE–Ca–Si–O–N* system (where *AE = Mg*, *Sr*, and *Ba*) were prepared from fine powders of *AEH_2_* purchased from Alfa Aesar GmbH & Co (Kandel, Germany) with >95.6% metal basis, *SiO_2_* purchased from ABCR GmbH & Co (Karlsruhe, Germany) with 99.9% purity, and *Si_3_N_4_* purchased from ChemPur GmbH (Karlsruhe, Germany). In order to study the effect of mixed modifiers of AE–Ca, the nominal composition of *Ca_26_Si_19_O_33_N_22_* was chosen. Five grams of each batch was melted in niobium crucibles using a radio frequency (RF) furnace. The samples were first heated up to 650 °C over 15 min and held at this temperature for 30 min to completely drive off hydrogen. The samples were then heated to 1000 °C over 15 min and held at this temperature for 30 min for complete reaction of the metal hydride with nitrogen, and finally heated in the temperature range 1600 to 1700 °C according to the composition of the glass. Subsequently, the melts were cooled to the room temperature by switching off the furnace at the end of each experiment. Nitrogen-rich glasses were prepared under N_2_ flow, serving as a nitridation source for metal hydride. The composition of each analyzed glass is given in Table 1. Cation concentrations were determined by energy-dispersive X-ray (EDX) point analysis (20 points on each sample) on polished and carbon-coated surfaces, using a Si detector and a LINK INCA program system. Oxygen and nitrogen contents were determined by combustion analysis using LECO equipment. Further descriptions regarding the synthesis of these nitrogen-rich glasses and their subsequent characterization are provided elsewhere [33].

The density of the bulk glass samples was measured in distilled water at room temperature using the Archimedes principle. Measured glass densities were reproducible to ±0.005 g/cm^3^. Molar volume (*Mv*) and atomic packing density values were calculated from the measured density and chemical composition of the glass. The micromechanical properties, e.g., (nano)hardness (*H*) and reduced elastic modulus (*Er*), of the bulk glass samples were measured by nanoindentation using a Nano Test Vantage instrument from Micro Materials (UK). SiC papers were used to polish glass samples to a mirror finish in ethanol, and the last step of sample polishing was performed using a polishing cloth in diamond suspension. A standard Berkovich diamond tip having a 142.3° total included angle and 65.35° half-angle was used to study the indentations of the prepared glasses. A load of 20 mN was used for indentation. Before each experiment, the Berkovich diamond tip was calibrated using a fused silica sample, and each sample was measured 14 times to reduce experimental errors. Oliver and Pharr’s approximation was used to calculate the hardness and reduced elastic modulus by measuring the unloading elastic part of the load–displacement curve [36].

The elastic properties of nitrogen-rich *AE–Ca–Si–O–N* (where *AE = Mg*, *Sr*, and *Ba*) glasses were measured by ultrasonic echography on polished glasses samples, at room temperature. A piezoelectric transducer (10 MHz), driven by shock excitation, and a pulse/receiver (Model PR 35, JSR Ultrasonics, New York, NY, USA) were used to generate the longitudinal and transverse ultrasonic waves. The following relations were used to estimate the elastic modulus:

(1) $$E=\rho \;\frac{\left(3V_{i}^{2}-4V_{t}^{2}\right)}{\left(\left(V_{i}^{2}-V_{t}^{2}\right)-1\right)},$$

(2) $$G=\rho V_{t}^{2},$$

(3) $$K=\frac{E}{3\left(1-2\nu \right)},$$

(4) $$\nu =\frac{E}{2G}-1,$$

where “ρ” is the density of glass, “$V_{i}$” is the ultrasonic velocity of the longitudinal wave, “$V_{t}$” is the ultrasonic velocity of the transverse wave, “*E*” is Young’s modulus, “*G*” is the shear modulus, “*K*” is the bulk modulus, and “*ν*” is Poisson’s ratio.

## 3. Results and Discussion

Table 1 and Table 2 summarize the composition and analyzed property values of the three series of nitrogen-rich mixed alkaline earth silicon oxynitride glasses. The nitrogen and modifier contents are expressed in terms of atomic percentage (at.%). The composition of the glasses in Table 1 corresponds to the same composition as in [33]. All the obtained glasses were X-ray amorphous as confirmed by X-ray diffraction (XRD) using an X’pert PRO diffractometer PANalytica, (Cambridge, UK) with CuKα radiation. The Mg-containing glasses had a high affinity toward nitrogen when compared to the Sr- and Ba-containing glasses. This was due to the lower radius of Mg ions; in general, the radius of ions also affects the N retention in the glass network. The Mg-containing glasses comprised up to 19 at.% N. Moreover, the variation of N content in the glasses was much smaller within the group of each series. Compositional analysis showed that the prepared glasses had lower contents of AE and N than the starting mixtures, and the Mg losses were more evident as compared to the Sr and Ba losses. Further details on the glass formation and the microstructure are provided elsewhere [33]. The Mg-containing glasses were more transparent than the Sr- and Ba-containing glasses, with less amount of metallic silicide as compared to the Sr- and Ba-bearing glasses. Previous reports on *M–(Al)–Si–O–N* glasses demonstrated that these glasses are nontransparent in the visible range [6,8,10,14,15,22,37,38,39,40]. The present glasses in the Mg-series were transparent to the naked eye, and the transparency increased with Mg content in the glass network. Generally, impurities in the form of metallic silicide and/or elemental Si particles presumably accounted for the nontransparent and gray/black glasses. The transparent glasses obtained in the Mg-series, thus, suggest that the presence of Mg in oxynitride glasses prevents the formation of silicides.

### 3.1. Effective Cation Field Strength, Bridging Oxygen, and Network Connectivity

**Figure 1:**
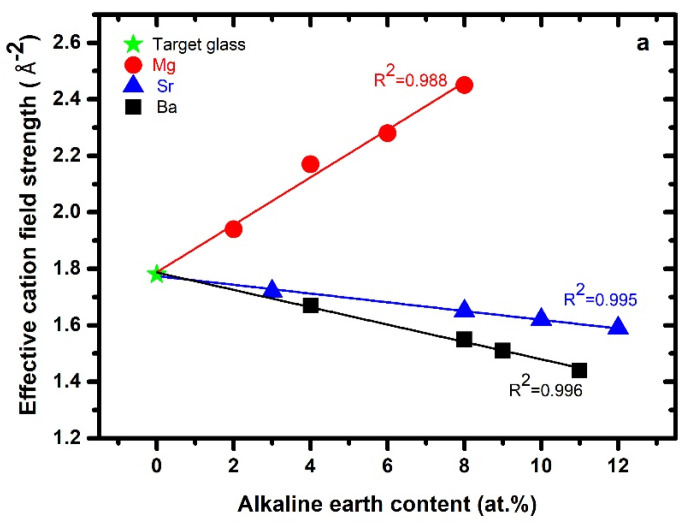

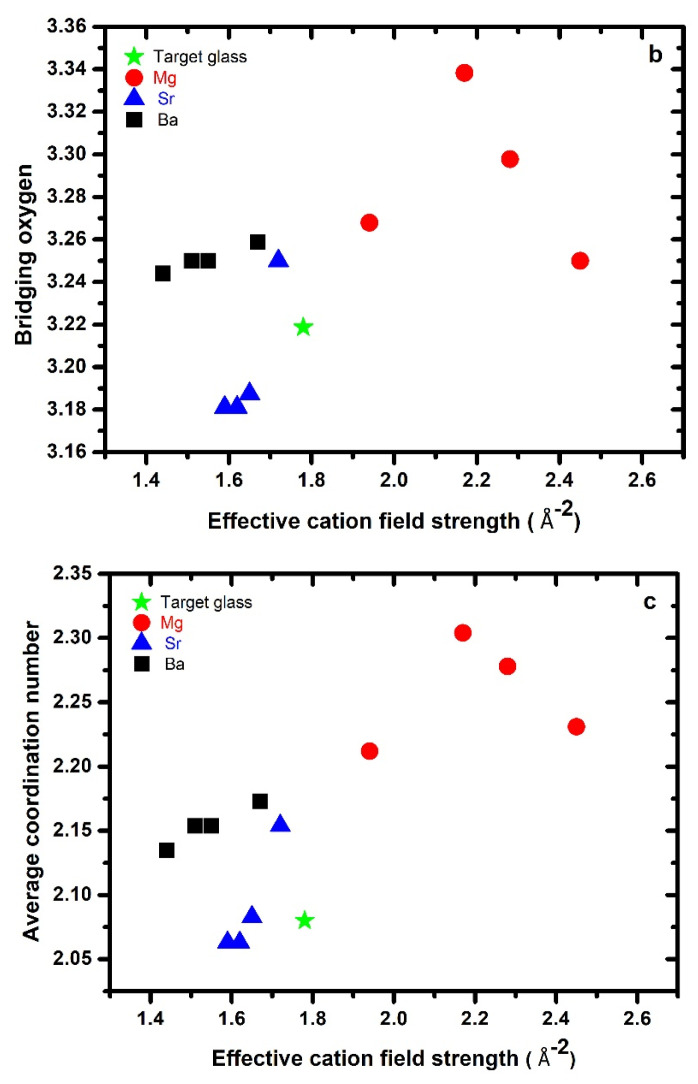
(**a**) Effective cation field strength plotted against alkaline earth content for each series; the lines represent the best-fit results with the as-indicated correlation coefficients involving both the target glass and all three series. (**b**) Bridging oxygen and (**c**) average coordination number as a function of effective cation field strength.

Calculated values of the effective cation field strength (*ECFS*) are given in Table 1. The *ECFS* was calculated as *ECFS = x_Ca_ × CFS_Ca_ + x_AE_ × CFS_AE_*, where *x_Ca_* and *x_AE_* are the atomic fractions of Ca and AE. The cation field strengths (*CFSs*) of the modifier ions were calculated as 3.858, 1.780, 1.366, and 1.050 Å^−2^ for Mg^2+^, Ca^2+^, Sr^2+^, and Ba^2+^, respectively, according to *CFS = Z/r^2^*, where “*Z*” is the valance of the respective alkaline earth metal, and “r” is the Shannon–Prewitt ionic radius for six-coordinated Mg and seven-coordinated Ca, Sr, and Ba. The variation of the *ECFS* as a function of the alkaline earth content is shown in Figure 1a. The Mg-series had higher values of *ECFS*, ranging from 1.94 to 2.45, which increased with Mg content in the series. The *ECFS* values of Mg-bearing glasses exhibited a linear fit with the Mg + Ca content (*R^2^* = 0.988). An increase in *ECFS* with Mg content was also found in *Ca–Mg–Al–O–N–F* glasses [41,42]. The Sr-series had ECFS values between 1.59 and 1.78 (*R^2^* = 0.995), which decreased with increasing Sr content. The Ba-series had ECFS values ranging from 1.44 to 1.78, which decreased linearly with increasing Ba content. This is supported by Figure 1a, which evidences a strong correlation (*R^2^* = 0.996) between ECFS and Ba + Ca content.

The values of the number of bridging oxygen ($n_{BO}$) atoms per glass-forming cation were calculated according to $n_{BO}=4-\underset{i}{\sum }M_{i}\;Z_{i}/(\underset{j}{\sum }\;F_{j})$, where $M_{i}$ is the atomic fraction and $Z_{i}$ the valency of the *i*-th modifying cation, and $F_{j}$ is the fraction of the *j*-th glass-forming cation. In the case of oxynitride glasses, the $n_{BO}$ number can be estimated by replacing [O] by an equivalent anionic concentration [O*], with [O*] = [O] + 3/2[N], assuming three-coordinated nitrogen in the glass network. For the Mg-series, the $n_{BO}$ values increased upon substitution of Mg for Ca up to 3.34 for glass Mg-2, while no systematic variation was observed for an increase in $n_{BO}$ with the *ECFS* of Mg-bearing glasses (Figure 1b). For the Sr- and Ba-series, $n_{BO}$ increased with the *ECFS* of Sr and Ba. Treating all the three series collectively shows that the number of bridging oxygen increased roughly with increasing *ECFS*. Dependencies on the modifying cation effect cannot be derived from this data; however, the $n_{BO}$ generally increased with nitrogen content, which resulted in the Mg-series having a high $n_{BO}$ in the glass network.

The average coordination number 〈n〉 was calculated according to the expression $\langle n\rangle =\frac{\left[4\times Si-2\times \left(Ca+AE\right)+2\left(O-\left(Ca+AE\right)\right)+3\times N\right]}{\left[Si-Ca-AE+O+N\right]}$, where *Si*, *Ca*, *AE*, *O*, and *N* represent their atomic concentrations in the glass. The values of <*n*> are given in Table 1 and plotted against *ECFS* in Figure 1c. The Mg glasses had high values of <*n*> as compared to the Sr- and Ba-bearing glasses. The correlation between *ECFS* and average coordination number manifested almost similar trend to that observed in the case of $n_{BO}$.

### 3.2. Density, Molar Volume, and Glass Packing Density

**Figure 2:**
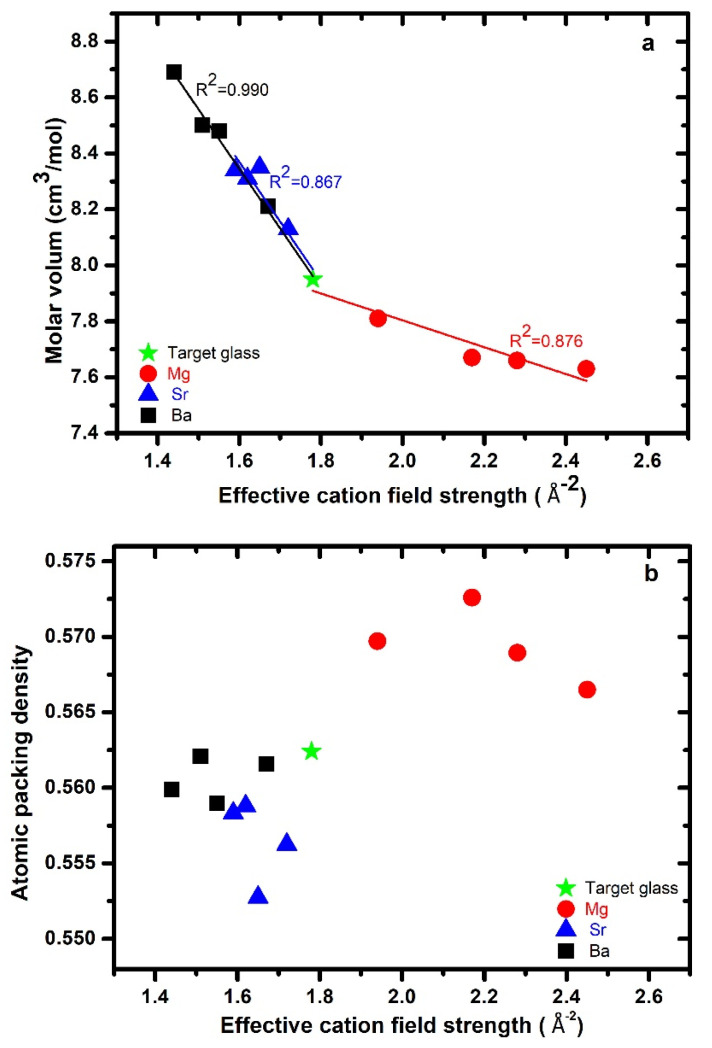
(**a**) Molar volume and (**b**) atomic packing density of glass as a function of effective cation field strength for each series (Mg, Sr, and Ba) along with the target glass.

The density (*ρ*) and molar volume (Mv) of these glasses were previously reported [33]; hence, only a short description is given here. The density of the Mg-series slightly decreased with increasing substitution of Mg for Ca. For the Sr and Ba glasses, the density increased with an increase in the substitution of Ca by Sr/Ba. Glasses in the Ba-series had a higher density compared to the glasses in the Mg- and Sr-series. The dependence of molar volume on *ECFS* is shown in Figure 2a. The molar volume increased as the larger and heavier Sr and Ba ions were substituted for Ca ions. When plotted versus the *ECFS*, the variations upon Sr and Ba substitution were quite similar, and the molar volume increased with decreasing *ECFS*. For the Mg-series, the trend was different, whereby the molar volume slightly decreased with Mg substitution for Ca. The general trend of the collective data shows that the molar volume decreased with increasing *ECFS*.

The atomic packing density/compactness ($C_{g}$) of the glasses was calculated according to the expression $C_{g}=\overset{\;}{\underset{\;}{\sum }}\left(X_{i}V_{i}\;N/Mv\right)$, where “*X_i_”* is the fraction of ionic species *i*, “*V_i_*” is the ion volume of the *i*-th element, calculated using the ionic radii given by Shannon [43], *N* is Avogadro’s number, and Mv is the molar volume. The glass atomic packing density as a function of *ECFS* is shown in Figure 2b. The Mg-series had higher values of $C_{g}$ (0.562–0.573) as compared with the target glass (0.562). The Sr-bearing (0.553–0.559) and Ba-bearing (0.562–0.571) glasses had lower values of $C_{g}$ than the target glass, thus suggesting a larger free volume in the Sr- and Ba-series.

In our previous study on *AE–Si–O–N* systems, we found that increasing the N content increased the atomic packing density of the glass, and the *Mg–Si–O–N* glasses showed higher values of atomic packing density as compared to the *(Ca/Sr/Ba)–Si–O–N*-containing glasses [17,22,39]. The different behavior observed for Mg is possibly due to Mg not acting as a modifier but forming part of the glass network [5,20,22]. $C_{g}$ and the glass network dimensionality are mostly interconnected; for example, metallic glasses have cluster-like structural units, corresponding to $C_{g}$ > 0.70. Oxynitride, low-Si-content silicate, and phosphate glasses consist of chain and layer units ($C_{g}$ > 0.55); conversely, oxide glasses mostly have $C_{g}$ < 54.

### 3.3. Hardness

**Figure 3:**
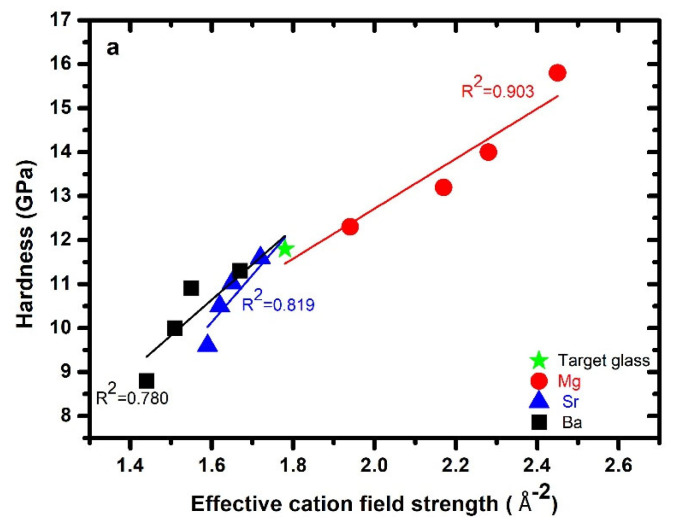

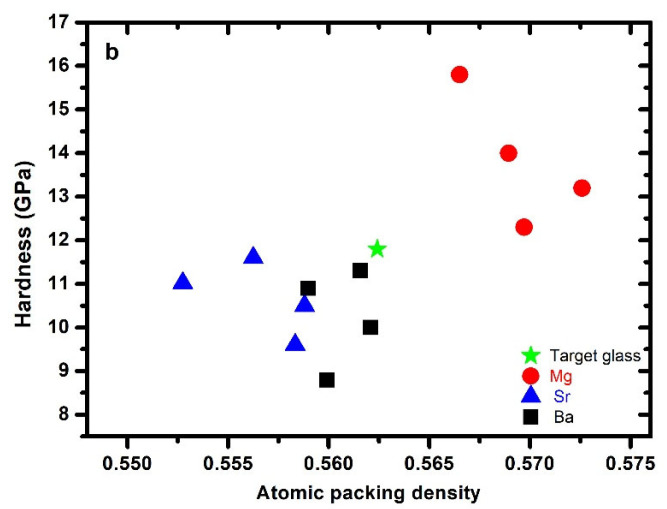
(**a**) Experimental hardness obtained by nanoindentation plotted against effective cation field strength, where the correlation coefficient indicates the linear fit involving both the target glass and all three series. (**b**) Hardness as a function of atomic packing density for each series (Mg, Sr, and Ba), along with the target glass.

The hardness (*H*) values for the high-nitrogen-content *AE–Ca–Si–O–N* glasses analyzed by nanoindentation are given in Table 2, including previously reported Vickers hardness (*H_V_*) values at a load of 300 g, and plotted vs. *ECFS* in Figure 3a. The data show that the effect of progressive substitutions of Ca for Mg, Sr, and Ba was nonmonotonic. The hardness values ranged between 12.3 and 15.8 GPa for the Mg-series, between 9.6 and 11.1 GPa for the Sr-series, and between 8.8 and 11.3 GPa for the Ba-series. In the case of the Mg-series, the hardness increased linearly with the Mg content, with *R^2^* = 0.903; in the case of Sr- and Ba-bearing glasses, the hardness decreased with increasing Sr and Ba substitution for Ca ions in the glass network (*R^2^* = 0.819 and 0.780, respectively). The current findings corroborate the previous reported Vickers hardness values for these glasses (Table 2). Hardness is frequently related to the *CFS* of the modifier elements, whereby glasses incorporating the highest *CFS* ions have increased hardness.

For the present glasses, the hardness increased linearly with increasing *ECFS*, corresponding to a strengthening of the glass network by an increase in strength of the *AE–O* bonds. Our previous study showed that glasses in the *Mg–Si–O–N* system had high hardness values as compared to the other *AE–Si–O–N* systems [15,22,39,44]; similarly, *Mg–Si–O–N* thin films [23,25] had higher hardness values than *Ca–Si–O–N* thin films [24] prepared by magnetron sputtering. The present results agree with those found for mixed *AE/RE* element-containing oxynitride glasses, for which the hardness increased with *ECFS* [19,45,46]. The data indicate that the hardness of the glasses exhibited an additional effect as a function of the AE/RE type and concentration, despite the modifiers providing weak bonding pathways for crack propagation. Nitrogen is well known for strengthening and tightening the glass network in silicate glass systems; furthermore, previous extensive studies have shown that hardness is more dependent on the N content as compared to modifiers, which causes depolymerization of the network by converting bridging oxygen ($n_{BO}$) into nonbridging oxygen ($n_{NBO}$) species. The hardness of a silicate-based oxynitride glass is also dictated by the atomic packing density ($C_{g}$) of the glass; generally, hardness increases with the atomic packing density of the oxynitride glass network. The gross trend between hardness and $C_{g}$ for the present mixed modifier oxynitride glasses is shown in Figure 3b. It is difficult to see a clear trend in the individual series, but the Mg-bearing glasses had a higher value of hardness than the target glass, while the Sr- and Ba-bearing glasses had a lower hardness than the target glass.

### 3.4. Elastic Properties

**Figure 4:**
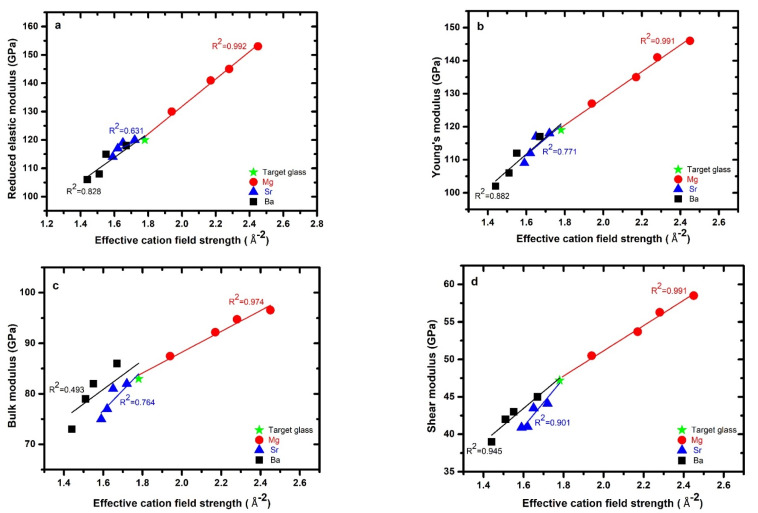
Left panel: Experimental values of the (**a**) Reduced elastic modulus obtained by nano-indentation, (**c**) bulk modulus and Right panel: (**b**) Young’s modulus (**d**) share modulus plotted against effective cation field strength, the lines represent best-fit results with the as-indicated correlation coefficients involving both target glass and all three series.

The reduced elastic modulus (*Er*) values of all three series measured with nanoindentation are given in Table 2, along with the elastic modulus (i.e., Young’s modulus (*E*)), shear modulus (*G*), and bulk modulus (*K*) values measured by ultrasonic echography. The reduced elastic modulus as a function of *ECFS* is plotted in Figure 4a, showing that it increased markedly with Mg substitution, by ca. 29%, with the highest observed elastic modulus being 153 GPa for the sample having the composition *Mg_8_Ca_16_Si_21_O_37_N_18_*. It decreased in the Sr-series by ca. 5% and in the Ba-series by ca. 12%. A similar trend was also observed for the Young’s modulus, shear modulus, and bulk modulus, which increased with the concentration of Mg in the Mg-series and decreased with increasing Sr and Ba content in the Sr- and Ba-series. The elastic modulus of amorphous materials depends on the strength of the bonds, i.e., in the Si (O,N)_4_ tetrahedra of the glass network and between modifiers (Ca and AE here) and the anions (O,N), as well as the packing state of the atoms. The bond strength between the anions and Si is stronger than that between the modifiers and O or N. Generally, the modifier contents (here, Sr and Ba) in the glass network cause depolymerization, i.e., disruption of the framework, and decrease the number of bridging oxygens, which leads to a decrease in the fraction of strong bonds between tetrahedra and, therefore, lower elastic modulus values. The relatively high values of elastic modulus for Mg–Ca silicon oxynitride glasses can be explained by the high bonding strength between magnesium and silicon tetrahedra. In oxide glasses, Mg acts a network modifier and is usually octahedrally coordinated by oxygen; in contrast, in nitride glasses, Mg is always tetrahedrally coordinated by nitrogen. In the case of oxynitride glasses, the coordination of Mg^2+^ is not known; it can be either octahedral or tetrahedral. Due to its high cation field strength, Mg can act as a network former in oxynitride glasses. Sr and Ba cations are known to act both as charge-compensators and as modifiers in oxide and oxynitride glass networks. As network modifiers, they cause depolymerization of the glass network by converting bridging oxygen ($n_{BO}$) into nonbridging oxygen ($n_{NBO}$). The Young’s modulus as a function of *ECFS* is shown in Figure 4b.

A similar trend was also observed for the bulk and shear moduli of these mixed-modifier oxynitride glasses, as shown in Figure 4c, and 4d respectively. In the case of oxynitride glasses, the bulk modulus is related to the bond density (number of bonds in a unit volume) and the bond stretching force, which are related to the cation field strength of the modifier cations [47]. Glasses in the Mg-series had higher values of both bulk and shear moduli than those in the Sr- and Ba-series. The bulk and shear moduli of these glasses both monotonically increased with *ECFS* for all three series. Sr- and Ba-bearing glasses had lower values of shear and bulk moduli than the target glass.

Furthermore, all three moduli (Young’s, bulk, and shear) are governed by the nitrogen content in the glass network, which generally produces a more tightly and highly linked glass network. The incorporation of N into the oxide glass network linearly increased the elastic modulus of the glasses [3,48,49,50].

### 3.5. Poisson’s Ratio

**Figure 5:**
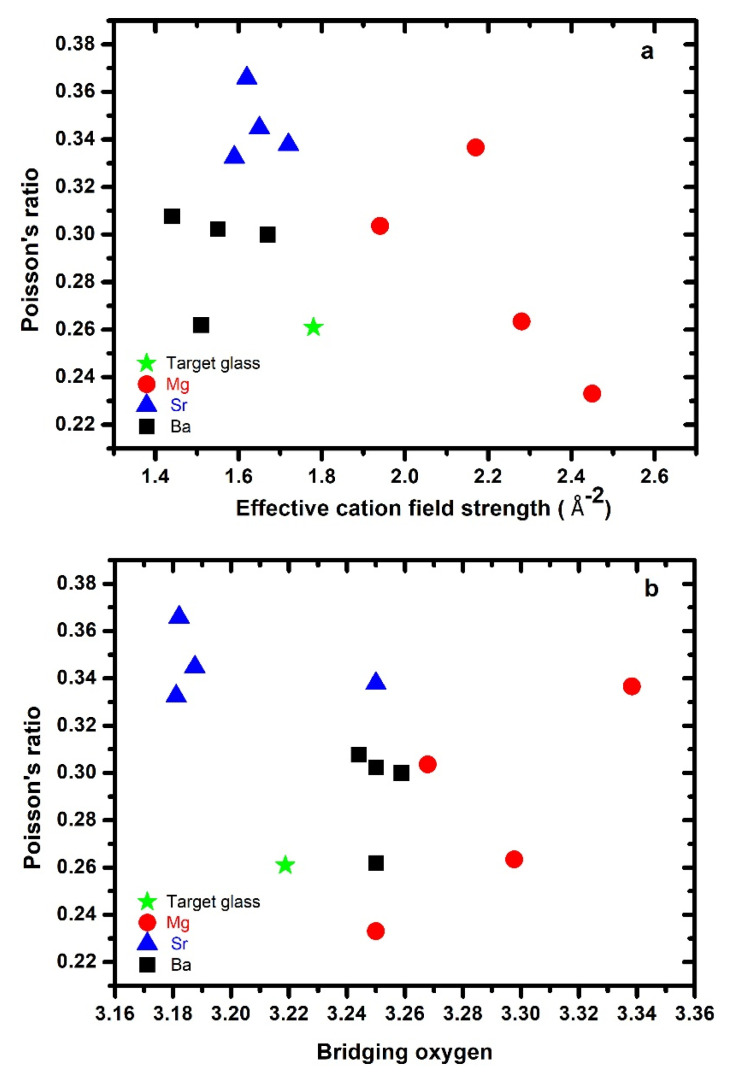

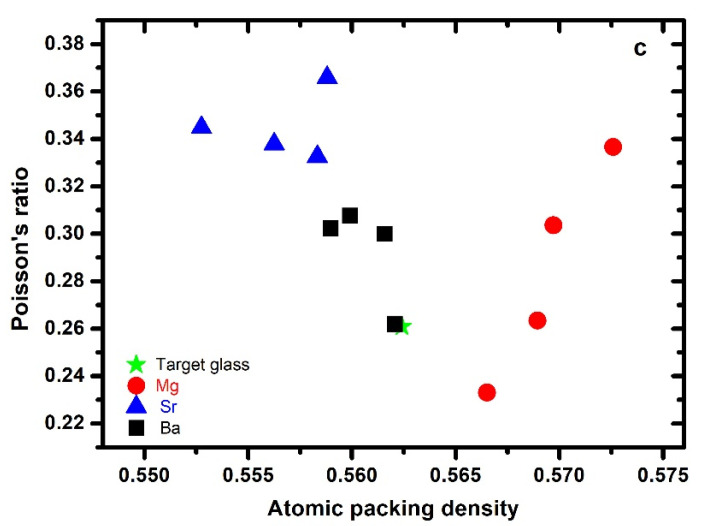
Poisson’s ratio plotted against (**a**) effective cation field strength, (**b**) number of bridging oxygens, and (**c**) glass atomic packing density for each series (Mg, Sr, and Ba), along with the target glass.

Figure 5a displays the variations in Poisson’s ratio with respect to *ECFS*. It is difficult to see any clear trend among the three series; however, Poisson’s ratios generally decreased with increasing *ECFS* of the glasses. The number of bridging oxygen atoms ($n_{BO}$) per glass-forming cation seems to be the key to understanding the Poisson’s ratio dependence of the oxynitride glasses. Compared to oxide glasses, oxynitride glasses often show high values of Poisson’s ratio. It must be noted that $n_{BO}$ does not vary monotonically with N content because of its dependence on modifier cations. In the present work, the Poisson’s ratio nonlinearly decreased with increasing $n_{BO}$ in the Sr-series. The opposite trend was observed for Mg- and Ba-bearing glasses (Figure 5b). For a similar number of bridging oxygen atoms, the Sr-containing glasses had high values of Poisson’s ratios. The higher values of Sr–Ca glasses might be due the presence of the high amount of silicide in these glasses. According to our previous study, the Poisson’s ratio of *Ca/Sr–Si–O–N* glass increased with the metallic silicide and elemental Si present in the glass network [14]. Both silicides and elemental Si are considered impurities in the glasses. Furthermore, it was observed that, as the average coordination number of network formers increased, the Poisson’s ratio decreased. However, the mechanisms behind this are still unclear and need further elucidation. No systemic variation was observed in the Sr- and Ba-bearing glasses; in the case of Mg-bearing glasses, the Poisson’s ratio increased with the atomic packing density (Figure 5c). Unfortunately, a direct prediction of Poisson’s ratio from the chemical composition remains challenging, particularly in the case of nitrogen-containing glasses.

### 3.6. Absence of Mixed Modifier Effects

The mixed alkali/alkaline earth effect, also called the mixed modifier effect (*MME*), occurs when two distinct alkali or alkaline earth metal cations are incorporated in the glass network. MME is one of the most interesting unsolved problems in glass science and technology, and a comprehensive study of the *MME* is needed. The obtained results showed nonadditive changes in the *MME* upon the substitution of one modifier for another at a constant total modifier content. The *MME* strongly manifests cation mobility/conductivity properties, nonadditive scaling of viscosity, hardness, glass transition temperature, and chemical durability. A relatively small or no deviation from linearity occurs for the density, refractive index, and elastic properties with *MME* [51,52,53]. In comparison with the mixed alkali effect, very little attention has been paid to the mixed alkaline earth effect. Previous numerous investigations of alkali and alkaline earth oxynitride glasses revealed the absence of a mixed modifier effect. Exceptionally, Wang et al. [54] very recently showed that *(Ca,Mg)–Si–Al–O–N* glasses prepared using the sol–gel method had a mixed modifier effect but only for the glass transition temperature, whereas no mixed effect was observed for the reported density, molar volume, Vickers hardness, and compressive strength. Furthermore, the authors only reported the nominal composition of the glasses; thus, the concentration of N in the glass network is unclear. Considering the current and previously reported nitrogen-rich mixed-modifier glasses, we did not observe any mixed modifier effects [9,31]. This is due to the fact that the variation in such properties was most likely due to the presence of nitrogen in the glass network rather than the modifier content. Furthermore, more work is required to probe the absence/presence of the *MME* for a wider range of compositions in Si-, B-, and P-based oxynitride glasses.

## 4. Conclusions

This paper provided new insights into the relationships between the composition and properties of nitrogen-rich mixed alkaline earth silicate oxynitride glasses. The *ECFS* of the as prepared glasses increased linearly with the Mg-series but decreased with the Sr- and Ba-series. The atomic packing density, number of bridging oxygens, average coordination number, hardness, and elastic moduli were enhanced when Mg replaced Ca in the silicon oxynitride glasses. In the Mg-bearing oxynitride glasses, N directly influenced the behavior of Mg, by favoring Mg cations in the tetrahedral sites and switching the behavior of Mg to a network-forming cation. On the other hand, the molar volume and Poisson’s ratio increased, while the atomic packing density, hardness, and elastic moduli decreased with increasing *ECFS* for both Sr- and Ba-series. Sr-bearing glasses had higher values of Poisson’s ratio than the Mg- and Ba-bearing glasses. The gross trend of all three series showed that the Poisson’s ratio decreased with increasing *ECFS*; however, the interpretation of the change in Poisson’s ratio within each glass composition presented a more complicated scenario without straightforward trend with the number of bridging oxygens and atomic packing density. No mixed modifier effect with alkaline earth metals was found in the as-prepared glasses, which is in agreement with previous findings in mixed-modifier oxynitride glasses; nevertheless, a more compositional dependence was exhibited.

## Figures and Tables

**Table 1:** Determined glass composition, effective cation field strength (*ECFS*), density (*ρ*), molar volume (*Mv*), atomic packing density ($C_{g}$), number of bridging oxygen ($n_{BO}$), and average coordination number 〈n〉.

*ID*	* *Glass Composition* (atomic %)	*ECFS* Å^−2^	# *ρ* g/cm^3^	# *Mv* cm^3^/mol	*C_g_*	*n_BO_*	〈n〉
Target glass	Ca_25_Si_19_O_40_N_16_	1.78	3.02	7.95	0.562	3.22	2.080
Series-Mg, Mg_x_–Ca_(25−x)_–Si_19_ O_40_N_16_							
Mg-1	Mg_2_Ca_22_Si_20_O_37_N_19_	1.94	3.02	7.81	0.570	3.27	2.212
Mg-2	Mg_4_Ca_18_Si_21_O_38_N_19_	2.17	3.00	7.67	0.573	3.34	2.304
Mg-3	Mg_6_Ca_17_Si_21_O_37_N_19_	2.28	2.98	7.66	0.569	3.30	2.278
Mg-4	Mg_8_Ca_16_Si_21_O_37_N_18_	2.45	2.96	7.63	0.567	3.25	2.231
Series-Sr, Sr_x_–Ca_(25−x)_–Si_19_ O_40_N_16_							
Sr-1	Sr_3_Ca_21_Si_20_O_40_N_16_	1.72	3.14	8.13	0.556	3.25	2.154
Sr-2	Sr_8_Ca_18_Si_19_O_37_N_18_	1.65	3.35	8.35	0.553	3.19	2.083
Sr-3	Sr_10_Ca_16_Si_19_O_38_N_17_	1.62	3.49	8.31	0.559	3.18	2.063
Sr-4	Sr_12_Ca_14_Si_19_O_38_N_17_	1.59	3.55	8.34	0.559	3.18	2.063
Series-Ba, Ba_x_–Ca_(25−x)_–Si_19_ O_40_N_16_							
Ba-1	Ba_4_Ca_20_Si_20_O_39_N_17_	1.67	3.35	8.21	0.562	3.26	2.173
Ba-2	Ba_8_Ca_16_Si_20_O_40_N_16_	1.55	3.67	8.48	0.559	3.25	2.154
Ba-3	Ba_9_Ca_15_Si_20_O_40_N_16_	1.51	3.84	8.50	0.562	3.25	2.154
Ba-4	Ba_11_Ca_13_Si_20_O_41_N_15_	1.44	3.99	8.69	0.560	3.24	2.135

* The composition of the glasses corresponds to the same composition as in [33]. Values might not add up to 100% due to rounding. # Values are taken from [33].

**Table 2:** The micro hardness (*Hv*), (nano)hardness (*H*), reduced elastic modulus (*Er*), Young’s elastic modulus (*E*), Shear modulus (*G*), bulk modulus (*K*), and Poisson’s ratio (*υ*).

*ID*	# *Hv* GPa	*H* GPa	*Er* GPa	*E* GPa	*G* GPa	*K* GPa	*υ*
Target glass	9.41	11.8	120	119	44.5	86	0.337
Series-Mg, Mg_x_–Ca_(25−x)_–Si_19_ O_40_N_16_							
Mg-1	10.48	12.3	130	127	48.7	94	0.304
Mg-2	11.33	13.2	141	135	52.5	98	0.286
Mg-3	12.01	14	145	141	55.8	110	0.263
Mg-4	12.16	15.8	153	146	59.2	117	0.233
Series-Sr, Sr_x_–Ca_(25−x)_–Si_19_ O_40_N_16_							
Sr-1	9.42	11.6	120	118	44.1	82	0.338
Sr-2	9.47	11.02	119	117	43.5	81	0.345
Sr-3	9.45	10.5	117	112	41.0	77	0.366
Sr-4	9.30	9.6	114	109	40.9	82	0.332
Series-Ba, Ba_x_–Ca_(25−x)_–Si_19_ O_40_N_16_							
Ba-1	9.47	11.3	118	117	45.0	86	0.300
Ba-2	9.31	10.9	115	112	43.0	81	0.302
Ba-3	8.92	10.0	108	106	42.0	79	0.261
Ba-4	8.29	8.8	106	102	39.0	73	0.308

# Values are taken from [33].

## Data Availability

Not applicable.
